# Applying Binder Jetting Technology to 316L Stainless Steel Materials and Testing Its Mechanical and Dimensional Properties Depending on the Printing Method

**DOI:** 10.3390/ma17174400

**Published:** 2024-09-06

**Authors:** Ján Kráľ, Tibor Dzuro, Hubert Debski

**Affiliations:** 1 Department of Production Technology and Robotics, Faculty of Mechanical Engineering, Technical University of Kosice, 042 00 Kosice, Slovakia; jan.kral.2@tuke.sk; 2 Department of Fundamentals of Machine Design and Mechatronic Systems, Faculty of Mechanical Engineering, Lublin University of Technology, 20-618 Lublin, Poland; h.debski@pollub.pl

**Keywords:** 3D metal printing, additive manufacturing, additive technology, binder jetting, metal parts, metallic powder, non-fused printing technology, powder metallurgy

## Abstract

This article discusses special additive technologies, with a particular focus on the innovative binder jetting technology used to create three-dimensional objects. The theoretical part of this article defines the production process–its shortcomings and benefits. Also, the article describes process parameters and individual steps that must be optimally set for the desired result. Further, the article characterizes the most influential factors that are indispensable in the printing process-metallic powder, binder, printing parameters, and finishing operations after the printing itself. The conclusion of the theoretical part deals with various material possibilities when using binder jetting technology. In the practical part of the article, the properties of the material, the chemical composition, and the resulting accuracy of the printed samples will be verified experimentally. The information obtained will subsequently be used to identify an economically advantageous application of binder jetting technology.

## 1. Introduction

Additive technologies make it possible to produce technologically unmanufacturable complex parts from various materials while reducing steps in the production process. Some additive technologies allow both design and production with excellent dimensional accuracy from materials such as polymers, ceramics, and metal [1].

Additive technologies represent production technologies whose principle is material layering in various ways, such as extruding, welding, curing, and others. As a rule, the process begins with the design of a three-dimensional (3D) object in a digital environment using CAD (Computer-aided design) systems. Using the machine and modeling program, individual layers and paths of the tool are then defined in order to achieve the desired geometry. In general, additive technologies are divided into two groups [2]:

1. Fusion-based methods—fuse-based principle (FBP):
  - DED—Direct energy deposition;
  - LPBF—Laser powder bed fusion;
  - EBM—Electron beam melting.
2. Non-fusion-based methods—no curing:
  - Extruding;
  - Binder jetting;
  - Lamination.

Depending on the material and the desired properties, individual processes differ slightly. Postprocessing of additive technologies can include finishing operations that are necessary to achieve the required tolerances.

The advantages of layer-by-layer production of components include a high level of freedom at the design stage, uniform production times, reduction of material waste through recycling, and the ability to create geometrically complex objects in a single operation. No rebuilding of the equipment is required for the production of another part; it is simply possible to manufacture object A alongside object B—if the capacity of the machine allows it. The nature of the technology predetermines its use also for the production of unique and complex parts, or for the production of prototypes, etc. By reusing unused materials, additive technologies are also suitable in sectors where scarce materials are used, such as aerospace.

Another advantage of additive technologies is that no specific tools are required for manufacturing, whereas conventional technologies, such as machining or forging, require a certain range of tools. Studies comparing the life cycle of additive technologies (especially binder jetting) and conventional technologies have shown that in addition to shortening production times, reducing steps in the production process, and minimizing tool needs, this technology also dominates in a more favorable environmental impact [3]. The mass adoption of additive technologies is currently hampered by several factors. The first is a smaller number of materials–especially metals and ceramics suitable for additive technologies. With regard to material properties, the relationships between process parameters and resulting properties have only recently begun to be characterized, resulting in lower confidence for structural applications. In addition, due to the novelty of additive technologies, machines and processes often differ, which also does not contribute to trust in the technology. The cost of additive technologies at this time is highly volatile due to the volatile prices of energy and input materials, which predetermines the use of additive technologies only in some high-value-added industrial sectors, such as aerospace and biomedical industries [4].

One of the methods of manufacturing components by additive technologies is the use of a powder bed, in which metallic powder is dispersed into a thin layer. This applied layer is subsequently glued together or joined together using a heat source. The joined layer is lowered, and another layer of powder is applied to it. The process runs until the printing of the entire part is complete. Just as additive technologies are divided, this method of manufacturing can also be divided into fusion technologies—those that use a heat source (laser, electron beam) and technologies that use a binder to bind layers—such as binder jetting [5]. It is this method of production that uses a binder to selectively bond the material in a powder bed. Ziaee and Crane [6] presented a broad review of technologies and approaches that have been applied in binder jet printing and pointed toward opportunities for future advancement. Li et al. [7] prepared a comprehensive review of the currently available literature sources on the jetting of metal binders. They summarized critical factors and their effects in the jetting of metal binders and divided them into two categories, namely material-related factors and process-related parameters. At the same time, they presented data on density, dimensional and geometric accuracy, and mechanical properties achieved by jetting a metal binder. By optimizing the parameters and choosing the sintering process appropriately, they showed that these materials achieve a relative density of more than 90%, with several types of stainless steel obtaining equivalent or better mechanical properties compared to cold forming. The above aspects require further scientific research, which includes understanding the powder coating process, binder-powder interactions, and shrinkage of parts. Shahed and Manogharan [8] investigated the powder-binder interaction for bimodal powders with actual binder jetting conditions over a wide range of packing densities and binder jetting conditions. Using Discrete Element Modeling (DEM—powder deposition)–VOF (Volume of fluid–binder interaction), they analyzed the interaction of powder and binder in terms of penetration depth, spreading time, and area per droplet on the powder bed. Their results provide a new understanding of the spatio-temporal characteristics of the binder-powder interaction, which helps in identifying the optimal printing parameters for the bimodal powder feedstock. Roberts et al. [9] investigated the maximum size of binder nanoparticles to fill the gaps between bed particles. Their results provide information on the selection of nanoparticle sizes required for binder composition, density optimization, and shrinkage reduction in jet binder printed components. Yang et al. [10] created a highly accurate computational model of liquid-solid interaction to simultaneously reproduce both binder flow and powder movement. The model accurately reproduces binder droplet impact, binder spreading and penetration, and powder particle spattering and agglomeration and provides in-depth insight into the fundamental mechanisms of binder-powder interactions as well as a basis for process and materials optimization. Mao et al. [11] developed an original binder and investigated the effect of layer thickness and binder saturation on the strength, dimensional accuracy, and surface roughness of raw 316L stainless steel samples produced by binder jetting additive manufacturing. They observed a decrease in strength and an increase in roughness with increasing layer thickness for raw printed samples. Increasing the binder saturation improved the strength and surface roughness of the raw samples. At the same time, they discussed the relationships between the binder and the mechanical performance of the raw material samples in terms of uneven distribution of the binder in depth. Yang et al. [12] simulated binder jetting and analyzed the joining mechanism of magnesium alloy. They used COMSOL Multiphysics simulation software (COMSOL 6.2) to create a simulation model of the binder movement and deposition process. They found that the higher the flow velocity, the larger the dispersion width of the binder drop after impacting the powder bed, which seriously affects the dimensional accuracy of the blank. Bae et al. [13] investigated the influence of binder and powder properties used in Binder Jet 3D Printing on Build-Up. They used an inorganic binder based on silicate (SiO_2_–Na_2_O) and powder in the form of sand. The results confirmed the increased print quality because the more hydrophilic the surface of the powder used for 3D printing, the higher the affinity between the surface and the inorganic binder, which increases the wettability. However, at the same time, the final strength of the product decreased. Lai et al. [14] investigated the effect of surface roughness on the fatigue behavior of 316L stainless steel produced by the binder jetting process. They performed Tension-compression fatigue tests on 316L stainless steel produced by binder jetting to assess the effect of surface roughness. Porosity was analyzed using an X-ray CT scan to study the distribution and the correlation to fatigue crack initiation. They found that the surface roughness lowered the fatigue strength by approximately 21%. The 316L material produced by binder jetting had lower yield strength than its fatigue strength.

This article deals with binder jetting technology for setting up mass production processes for 316L steel components. The properties of the material, the chemical composition, and the resulting accuracy of the printed samples will be verified experimentally. The information obtained will subsequently be used to identify an economically advantageous application of binder jetting technology.

## 2. Additive Binder Jetting Technology

**Figure 1:**
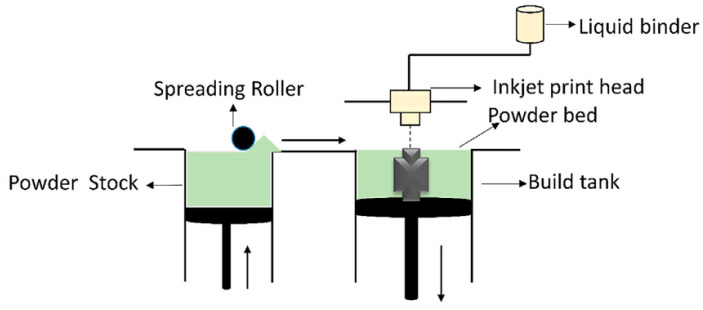
Schematic representation of the binder jetting [15].

**Figure 2:**
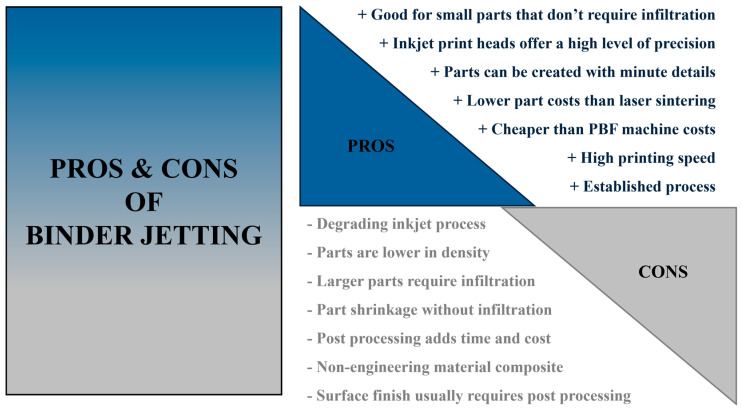
Advantages and disadvantages of binder jetting technology.

Binder jetting (Figure 1) is an additive manufacturing technology that uses a binder to selectively (Figure 2) bind powder particles together, creating three-dimensional objects layer by layer. This technology is highly versatile and can produce objects from a variety of materials, including metals, ceramics, sand, and polymers. As the process progresses, layers of material are glued together to the desired geometry. After layering is completed, if necessary, the entire container is placed in the furnace, where hardening and settling of the binder takes place. The temperature and time in the oven depend on the binder used. After this step, the excess unbonded powder is sucked out and can be reused. Depending on the material, some components are finished at this stage and do not need any finishing operations. Furthermore, metallic and ceramic materials need to be subjected to heat treatment, such as sintering or infiltration, using an additional material to achieve the required mechanical properties [5].

The most significant factor in binder jetting technology, currently limiting its potential, is the inability to estimate the level of deformations that occur during the sintering process. Recently, therefore, research has been focused on computational tools that minimize this problem [16]. Due to the similarity of technology with powder metallurgy and sintering, it is possible to use a wealth of knowledge directly from these sources. First of all, research is needed in the field of powder morphology, particle sizes, chemical composition, and their effects on the printing process itself. Other important parameters that affect density, strength, and other properties are the phase of the interaction of the binder and the powder, the thickness of the powder layer, the level of soaking of the binder, the drying time of the binder, and the printing speed. Post-processing operations, thanks to which we can achieve the required levels of density and surface quality, play an important role in the final properties and overall usability of products. In short, in addition to the knowledge drawn from powder metallurgy, casting, and other additive technologies, a number of studies are needed to optimize the process and further specify its parameters [5].

**Figure 3:**
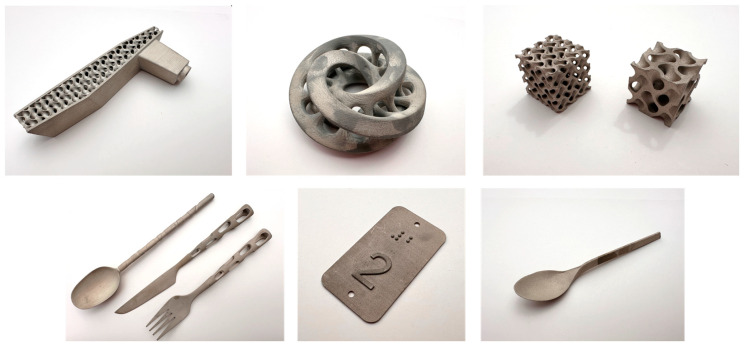
Examples of components made by binder jetting technology.

The following Figure 3. shows and describes examples of components made by binder jetting technology.

### 2.1. Processes in Binder Jetting Technologies

Processes in binder jetting technology include the following steps:

- Press

The current process of binder jetting is basically the same as it was at the birth of the technology. The fundamental change is mainly represented by new materials. ISO (International Organization for Standardization)/ASTM (American Society for Testing and Materials) 52900:2015 defines binder jetting as an additive manufacturing process in which a liquid binder is selectively applied to bind the powder into the desired shape [17].

To complete the geometric shape of a component, it is necessary to pay attention to the following steps [18]:

- A 3D model of a scanned, real, or digital object is required to create a CAD model. The model is digitally divided into layers and saved as an STL (Standard Triangle Language) file that is compatible with the machine;
- A thin layer of powder is spread in the container with a cylinder, which fills a thin gap and, with repeated opposite movements, pushes the excess powder away;
- A liquid binder (most often of polymeric origin in solvent or aqueous solution) is applied by the print head to a layer of powder at the points forming the geometry of the object [18]. Important is its ability to saturate into powder;
- After applying the binder, an electric heater passes through the powder container, which partially hardens and dries the binder. The heater also helps to maintain an even process temperature. The curing time after the application of individual layers is an important factor, as if the binder is insufficiently dried and bonded with the powder, cracking or sticking to the spreading roller could occur;
- After applying and drying the binder, the piston under the powder bed drops by a height of one layer (usually 50 to 200 μm). A further batch of powder is then prepared from the powder reservoir, which is spread, gently compacted, and smoothed to the correct height [5,19].

- Powder curing and suction

After printing is completed, some of the binder jetting technologies require additional operations to completely dry the binder, due to which the part acquires the required strength. Usually, the entire container is manually moved to the oven, where it is heated to a temperature between 180–200 °C. In the future, there will be an effort to automate this step. The curing time and conditions are derived from the binder properties and container volume. After passing this step, the parts have sufficient strength to move to the furnace. Before starting sintering, loose powder is removed with a vacuum cleaner. Depending on the complexity of the parts, manual cleaning with a brush or compressed air may also be required. Caution should be exercised when handling geometrically complex parts containing elements such as fine or overhanging parts. The cleaned part is then sintered or thickened with another material that enters the structure and provides the required mechanical properties [5].

- Sintering or infiltration with other material

The part has a relative density of 50–60% after the curing and powder removal phase. When observing the structure with a microscope, one can see individual grains of powder. In order to achieve the required density and mechanical properties, some of the methods already mentioned can be used. Regardless of the method of compaction of the structure, complete evaporation of the binder must first occur [5].

When choosing the right post-processing operation, it is important to start from factors such as material composition, powder particle size, sintering atmosphere, process time, and temperature [20]. As the characteristics of individual materials vary during sintering, it is sometimes advisable to adjust the process by using additives that help improve the compaction process. This can be achieved, for example, by mixing powders of different sizes or by using particles coated with different films [21]. Strategies differ from material types. Since ceramic materials have considerably higher sintering temperatures and lower densification potential compared to metals, metal infiltration of the ceramic matrix is a frequent combination to achieve the desired structure. On the other hand, when printing polymers that have considerably lower melting points, the polymerization of the structure occurs already when the individual layers are applied [22,23].

Depending on the material and the technology chosen, e.g., infiltration, a high degree of tolerances and accuracy can be achieved. Conversely, when sintering the entire volume of a part of some alloys, significant deformations occur. Therefore, it is important to consider the size and location of critical elements at the design stage. A factor that must not be overlooked is gravity, which can be the source of sinkholes formed during sintering. The correct orientation of the component is, therefore, also important [5].

- Finishing operations

A factor that directly affects the quality of the surface is the softness of the powder and the thickness of the layer. The thicker the layers and the larger the powder particles, the rougher the surface. However, very fine powders may become more difficult to apply due to their adherence to the dispenser. Uneven deposition of the layer can lead to an inhomogeneous structure in terms of the resulting density. From an economic point of view, it is more advantageous to print thicker layers, where the printing process is shortened [24].

### 2.2. Factors Affecting the Binder Jetting Process

As with other 3D printing technologies, there are several parameters that have a major impact on the properties of parts. They can be divided into the following groups [25]:

- Characteristics of powder

The characteristics of the powder have a major impact on the final product, process conditions, and economic aspects. A lack of knowledge and understanding of powder process and mechanics can greatly affect the resulting quality and cost of production. The most important features that are monitored in binder jetting technology include [26]:

- Geometry and morphology of the powder–most commonly defined by circularity;
- Powder spreadability and powder flow (defined by the Hall flow test);
- Mean particle size and PSD (Particle Size Distribution);
- Powder layering density;
- Surface and internal chemical composition.

Since binder jetting is a relatively new technology, it is not possible to directly apply all knowledge from other powder technologies (e.g., PM (Powder metallurgy) or MIM (Metal Injection Molding)) [27]. The methods of powder production also differ. Alignment and understanding of process requirements and materials engineering can open up further possibilities for product performance improvements and production improvements [28]. The nature of the binder jetting process makes it possible to maintain certain phases in particles that would be lost in fusion technologies. However, further research is needed into recycling and particle reliability, possibly developing new types of powders that would allow special properties to be achieved [29]. Table 1 describes the metallic powder materials currently in use.

An important parameter of the process is viscosity. It depends not only on the reliable dosing of the binder but also on the resulting accuracy and quality of surfaces. The suitability of the binder for the process can be derived using the Weber (We) and Reynolds numbers (Re). The Formula (1) after modification looks like this [5]:

(1) $$Oh=\frac{\sqrt{We}}{Re}=\frac{\eta }{\sqrt{\gamma \cdot \varsigma \cdot d}};.$$

where

*Oh*—Ohnesorg number;
*γ*—surface tension;
*ς*—density of the binder in the liquid state;
*d*—the diameter of the drop coming out of the nozzle;
*η*—dynamic viscosity of the binder in the liquid stat

- Print parameters

Print parameters can be categorized as follows:

- Print thickness;
- The print height is a parameter defined as the thickness of the binder-wetted powder layer in the direction of the *Z*-axis [30];
- Print and powder application speed;
- A lower speed leads to higher accuracy and homogeneity of the structure but negatively affects the length of the process. On the other hand, at higher speeds, the risk of cavities and irregular structures increases [31];
- The ability of the binder to saturate powder particles—Saturation;
- The ability to correctly dose the binder is directly dependent on the capacity of the print head and the arrangement of the individual nozzles located in it. Based on this, the level of saturation of the powder with the binder and the overlap of the binder between the rows are defined. Incorrect deposit of binder negatively affects the homogeneity of the structure as well as the dimensional inaccuracy of prints [32,33]. Similar to the speed of application of powder layers, the optimal setting of binder dosing is the result of trial and error by the user [5]. The wettability level can be approximated using Equation (2):

(2) $$Uz=\frac{1000\cdot V\;}{\left(1-\left(\frac{PR}{100}\right)\right)\cdot X\cdot Y\cdot Z\;};$$

where

V—volume of one drop of binder;
*PR*—powder density (powder bed density) [%];
X and Y—distances between nozzles [μm];
Z—coating thickness [μm].

- Drying time and heating element performance;
- The curing time depends on the selected saturation level, the type of binder and its chemical composition, the thickness of the layer, the wettability of the powder, and the properties of the powder bed (thermal conductivity, density, area, etc.);
- With no or short drying time, there is an increased risk of blockage of some parts of the print head, which is ultimately reflected in the properties of the component [33,34].

- Print orientation

For additive technologies, there are generally two terms related to print orientation that need to be defined, namely:

1. Layering orientation

It affects the porosity and mechanical properties of the component. In a study focused on print orientation, it was shown that samples printed at an angle of 45° had a compressive strength of 13.4 ± 4.6 MPa and a porosity of 37 ± 2%, while samples printed in a perpendicular direction showed a tensile strength of up to 45.1 ± 6.8 MPa and a porosity of 30 ± 2%. In another study on the influence of orientation on properties, it was reported that layering in the direction parallel to the pressure is approximately 48% stronger than in layers oriented in the direction perpendicular to the load [16];

2. Component orientation

The orientation of the component during printing also has a significant influence. It has been proven that the highest quality and accuracy is achieved when pushed to the primary plane *XY*. When printing primarily *Z*-oriented, components achieve lower surface quality and lower density in the raw state. This is due to the need for more layers compared to parts whose printing is oriented in the direction of the *X* or *Y* axis. Thus, it is clear that defects associated with orientation form primarily in weaker bound layers [35].

- Post-processing operations

After completing the printing stage, it is necessary to perform additional operations that will allow to achieve the desired properties. This includes [4,5,18,22,29,36,37]:

1. Desiccation/initial curing

The purpose of this step is to remove excess binder after the printing process. It usually takes place in a whole container with a printout and excess powder. In some methods, only the raw component is cured. The curing time and temperature depend on the binder used, geometry, wall thickness, component volume, and overall container size. The curing process ensures that the bonds between powder and binder are strengthened;

2. Removing unused powder

Removing unused powder from the printed part is necessary to achieve the required accuracy and purity of the resulting object.

3. Check the strength of the raw print

We consider a raw component to be a part that has undergone a curing process but has not yet undergone a compaction process by infiltration or sintering. Strength at this stage is very important, especially due to the manipulations when removing the remaining powder. If the strength of a raw component is too low, it will usually be damaged. Conversely, if a component is too durable, this may indicate an excessive amount of binder. Based on this, when setting process conditions, for example, in the case of a new type of powder or binder, the raw strength of the component is taken as a decisive parameter for proper process optimization. Since there is no prescribed standard for the strength control of a raw part for binder jetting technology yet, it is usually based on ASTM and MPIF standards;

4. Pyrolysis or firing of binder

Following the curing of the raw components, the binder firing step follows. This action takes place before the process of sintering or infiltration, usually in the same furnace. In order to achieve the right level of sintering or infiltration, it is necessary to remove the polymer binder that remains in the interparticle space. Most often, pyrolysis is used for this, in which thermal decomposition of organic materials occurs without the presence of oxygen-containing media. Firing occurs when heated above the decomposition temperature of the polymer binder or by reaction with gaseous components present;

5. Control of the content and impact of residues

The decomposition of the binder and the associated processes can result in the formation of residual carbon and oxygen. Residual substances most often form oxides in the case of oxygen and carbides in the case of carbon, which can negatively affect the properties of materials. The most important thing is to check for materials that are sensitive to even small changes in the content of certain substances, such as steel and carbon. Knowledge of the process and its optimal setting can have a significant impact on the final characteristics of products. If the binder firing process is properly managed and thus a minimum of impurities remains in the structure, a negligible negative effect on shrinkage can be expected;

6. Infiltration

This process involves the densification of the porous structure with a material with a lower melting point than the base material–such as steel and bronze. Thanks to this process, it is possible to achieve complete structure density with minimally affected dimensional accuracy. The driving force of the infiltration process is capillary phenomena. Infiltration in binder jetting technology favorably affects the mechanical properties of the final product, including hardness, tensile elasticity, yield strength, and others. Process optimization is essential to achieve the desired properties. Too high a level of infiltration can negatively affect dimensional accuracy. An insufficiently saturated base material has an inhomogeneous structure and worse mechanical properties. One of the innovations for achieving even better mechanical properties and structure is the process of infiltration in a vacuum;

7. Sintering

Sintering is another method of thickening a porous structure after printing. The sintering kinetics and structure thickening process depend on the chemical composition and surface characteristics of the powder, powder morphology, PSD, and sintering atmosphere. The most important aspects in binder jetting metal powder printing that affect shrinkage linearity, microstructure, structure phase formation, and porosity are PSD, morphology and chemical composition of the powder, heating parameters, cooling parameters, temperature, and sintering time.

### 2.3. Suitable and Researched Materials for Binder Jetting Technology

Binder jetting technology has a wide range of possible materials that can be used. Some of the most commonly used materials are, for example:

- Metals

Metals such as aluminum, stainless steel, and titanium, as well as various metal alloys, are popular for their high strength and durability [30,31]. Binder jetting makes it possible to create complex metal parts with relatively low production costs [38];

- Ceramics

Ceramic materials such as aluminum oxides, silicon oxides, and carbides are used in applications that require high temperature resistance and chemical stability [6]. Binder jetting allows you to create detailed and precise ceramic parts with complex geometries [38];

- Polymers

Various types of plastics and polymers, such as nylon, polycarbonate, and elastomers, are suitable for the production of prototypes, models, and functional parts. These materials are often affordable and have a wide range of properties [5,6];

- Composites

Composite materials that combine different types of fibers and matrixes are used to produce high-strength and lightweight parts [39]. Binder jetting allows you to create composite parts with precise fiber orientation and optimized properties [6,40].

## 3. Used Material and Equipment in the Practical Experiment

### 3.1. Used Material

In the experiment, 316L stainless steel powder was used, which is a low-carbon alloy known for high corrosion resistance and relatively good strength. In addition, it also has good formability and thermal properties within the 3D printing process. The material specifications given in Table 2. are directly provided by the ExOne printer manufacturer (Huntingdon, PA, USA). In Section 4, some properties of the material mentioned are verified through tests.

The chemical composition of the 316L steel material is described in the following Table 3.

The influence of parameters such as temperature, thickness of powder, glue and others have a major impact on the resulting shrinkage rate. These enumerated effects were assessed on the optical device KEYENCE IM-8000 (Osaka, Japan) (see Section 4.2).

### 3.2. Equipment Used

For experimental purposes, a device from ExOne was selected and subsequently used, namely the model DesktopMetal X160Pro^®^, on which all samples were printed.

The X160Pro^®^ (Gersthofen, Germany) provides a proven 3D printing method using binder jetting technology, capable of printing ultrafine metallic, sand, ceramic and specialty material powders. This is the largest device of this type, which has a capacity of up to 160 L. This device can be used for a wide range of production, whether it is prototyping or mass production. The greatest benefit of the device is the free hand at the design stage and the ability to easily produce even shape-complex parts for a single operation [42].

In addition to the machine itself, other parts are also necessary for the operation of the technology, such as the powder hopper, the furnace in which the binder solidifies, the powder removal device, and the sintering furnace.

After the binder has hardened and sufficient rigidity of the prints has been achieved for their handling, the platform is moved from the furnace to the equipment where the primary dedusting process takes place. In this step, the unsaturated powder is sucked out.

The working area of the machine is closed during the dedusting process–this is a dust-free process. The inside of the machine is equipped with an extraction head, the pressure of which is adjusted so as not to damage the integrity of the green parts. However, damage may occur with complex parts.

The stay of the parts in the sintering furnace ensures complete bonding of the material. The time during which the parts are in the furnace depends entirely on the material, powder, and binder. For 316L steel, the so-called 12-h recipe was used, which consists of heating to a sintering temperature of ≈1380 °C, holding at this temperature for 130 min, and then cooling to 22 °C. Due to the laminar heat flow in the furnace, the manufacturer states that the difference between the different zones is ±1 °C. The parts are inserted into the furnace chamber on special inserts.

## 4. Practical Experiment—Optical Measurement of Samples and Material Tests

### 4.1. Print Parameters

Even before the experiment itself, first of all, it is necessary to set the parameters of the press as well as the powder used. The following Table 4 describes the parameters of printing and powder used.

PSD refers to the particle size distribution in a given material. PSD is important because it affects material properties such as flow, density, surface area, and reactivity.

D10, D50, D90

These parameters are quantile values that describe the particle size distribution within the PSD.

- D10: Provides information about the smallest particles in the mixture. It is important for applications where fine particles can affect the reactivity or flow resistance of the powder. This value means that 10% of the particles in the powder have a diameter smaller than or equal to this value;
- D50: Also known as the median particle size, it indicates that 50% of the particles have a diameter smaller than or equal to this value. It is the median value of the particle size distribution. D50 is often used to assess the overall average particle size in the powder. In the case of 3D printing, it can affect the quality of the printed product and the homogeneity of the material;
- D90: Provides information about the largest particles in the mixture, which can be critical for mechanical properties or for ensuring a uniform surface appearance. This value means that 90% of the particles have a diameter smaller than or equal to this value.

The device allows you to adjust the ratio between new powder and recycled powder obtained in dedusting processes.

**Figure 4:**
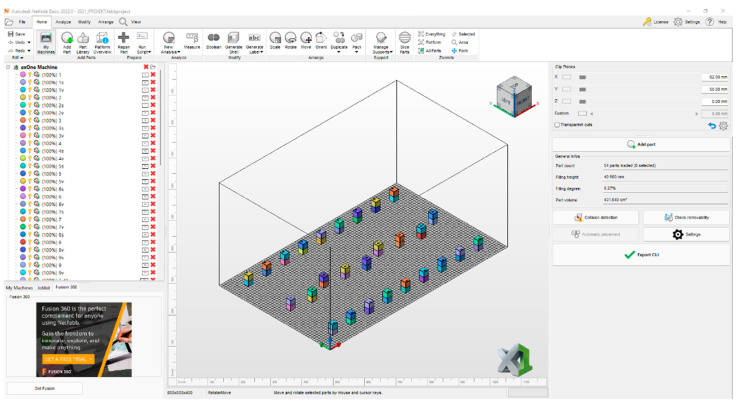
The work area of Autodesk Netfabb.

In Figure 4, one can see the arrangement of the cubes during the printing process. In addition to scrolling, printed parts can also be rotated freely. The correct orientation of the parts has a significant influence on the resulting deformation.

### 4.2. Estimation of Shrinkage in Individual Directions

**Figure 5:**
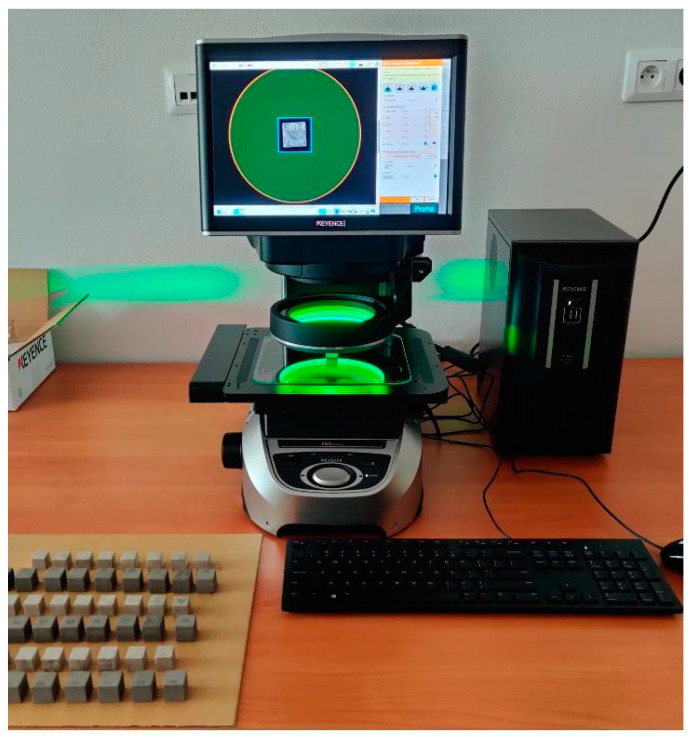
Measuring device KEYENCE IM-8000.

**Figure 6:**
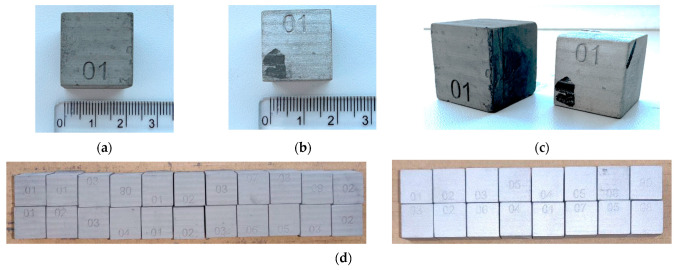
Printouts of test samples. (**a**) cube before sintering, (**b**) cube after sintering, (**c**) isometric view, (**d**) test samples of 316L.

Measurements were performed on a KEYENCE IM-8000 device (Figure 5). The test samples for measuring dimensions and shrinkage after sintering have the form of a cube with an initial side dimension of 20 mm (Figure 6a–c). Test specimens of 316L material are shown in Figure 6d.

**Figure 7:**
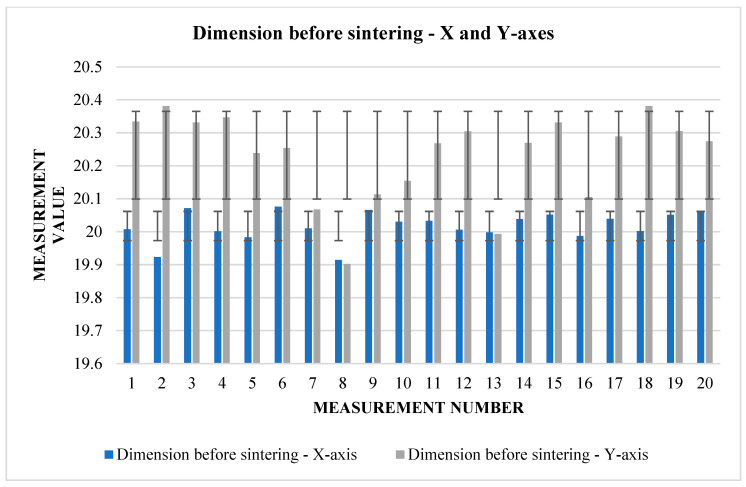
Graphical representation of Table 5—dimension before sintering—*X* and *Y*-axes (upper base).

The following Table 5 and Table 6. present the results of measurements of the first samples in different directions. These are cubes that have been pushed to the starting size of a 20 mm raw component, but this can vary from one direction to another due to factors such as gravity or printing inaccuracy. The measurements were carried out in three views (1, 2, 3), according to which we find and compare the relative shrinkage in the direction of the *X* and *Y* axes (Figure 7) in the upper and lower bases of the cube and in the direction of the *Z* axis in the side wall.

**Figure 8:**
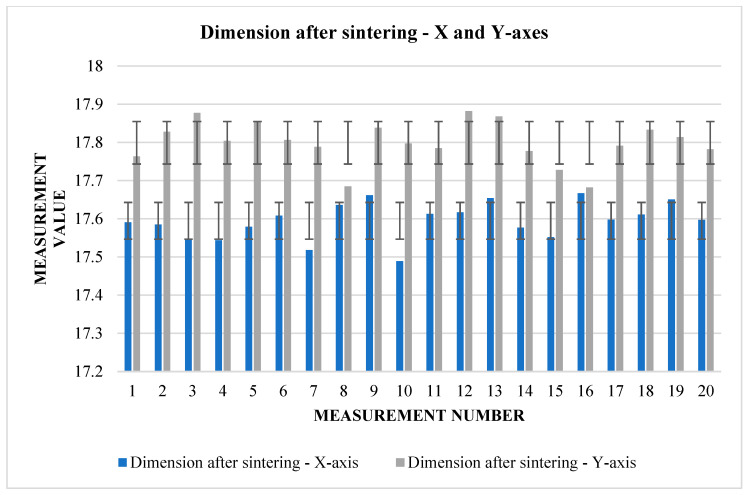
Graphical representation of Table 5—dimension after sintering—*X* and *Y*-axes (upper base).

From Table 5, it can be read that the change in dimensions after sintering in the directions *X* and *Y* (Figure 8) is similar at the upper base. In the direction of the *X*-axis, the average shrinkage was 2.423 mm. The percentage is 12.1%. In the direction of the *Y*-axis, the average shrinkage is similar—2.433 mm. As a percentage, it is 12.02%. However, the difference can be seen in the values of the dimensions before sintering between the two directions. In the direction of the *X*-axis, the average value measured on the device is 20.017 mm, while in the direction of the *Y*-axis, we measured an average of 20.232 mm. The shrinkage ratio after sintering is almost identical.

When measuring the lower base of the printed cubes (Table 6), we can observe a change in dimensions from the opposite base already before sintering. This can be caused by the combination of gravity factors and layering of the binder between the individual layers of the powder, where the binder can “overflow” along the edges of the cube. As for shrinkage, in the direction of the *X*-axis it is almost identical to the upper base. In the *Y* direction, it differs by two-tenths of a millimeter.

**Figure 9:**
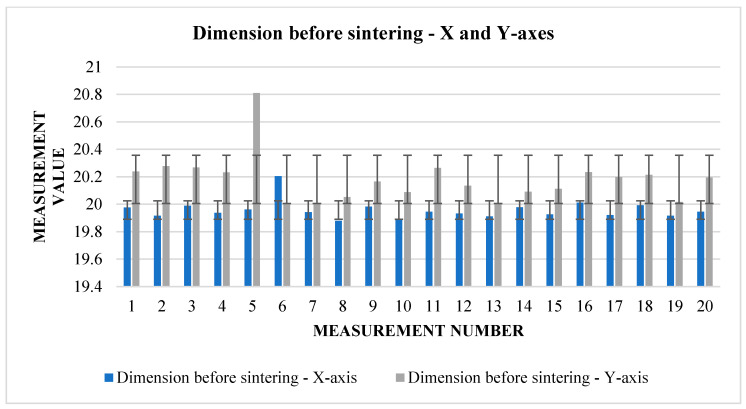
Graphical representation of Table 6—dimension before sintering—*X* and *Y*-axes (lower base).

**Figure 10:**
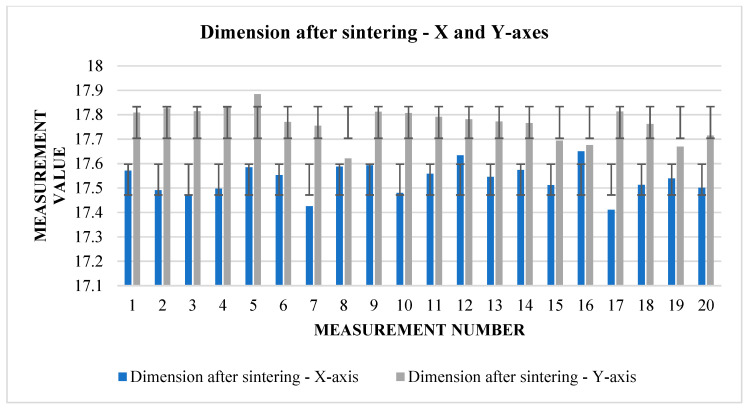
Graphical representation of Table 6—dimension after sintering—*X* and *Y*-axes (lower base).

**Figure 11:**
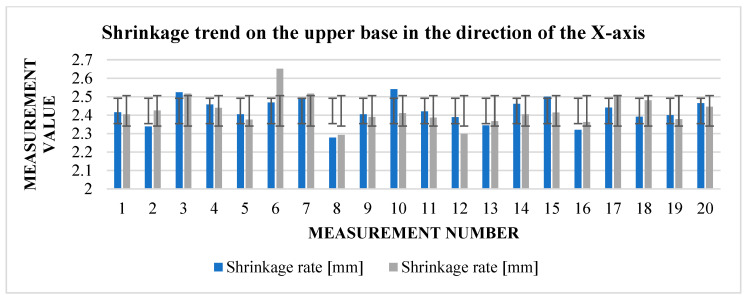
Graphical representation of Table 5 and Table 6—shrinkage in the direction of the *X*-axis.

**Figure 12:**
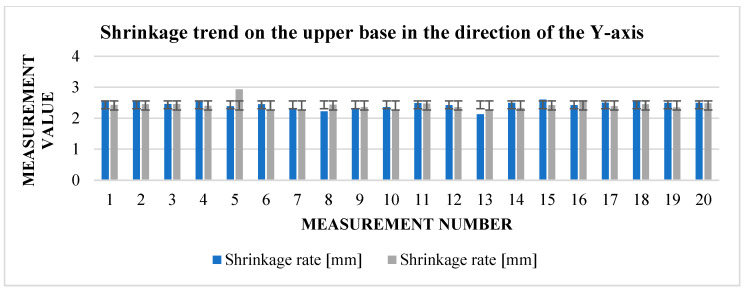
Graphical representation of Table 5 and Table 6—shrinkage in the direction of the *Y*-axis.

The graphs (Figure 9 and Figure 10) indicate some variation between individual cubes in both *X* and *Y* directions. There are also considerable rebounds between dimensions on both axes (Figure 11 and Figure 12), the origin of which is not yet known.

**Figure 13:**
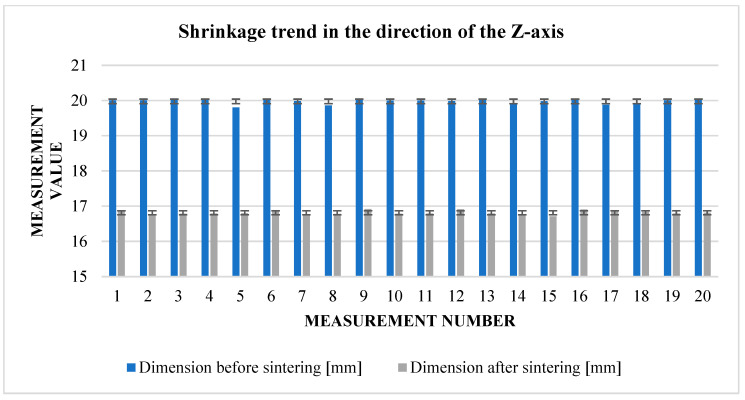
Graphical representation of Table 7—*Z*-axis shrinkage.

From the measured values obtained in the vertical direction, it is possible to immediately see the influence of gravity and the weight of the powder on the values of the resulting shrinkage. Compared to area shrinkage (*X*, *Y*), vertical shrinkage (*Z*) (Figure 13) is more than 3% higher (Table 7).

Based on the results obtained, we can estimate the rate of shrinkage and, thus, the need for additions to achieve the required dimensions in individual directions at approximately *X*: 12%, *Y*: 12%, and *Z*: 15.5%.

Thanks to this measurement, we have approached the shrinkage of cubes in different directions and the influence of gravity on printing accuracy, which will benefit the dimensional accuracy of future prints.

### 4.3. Optical Measurement of Samples with Set Shrinkage Rate

**Figure 14:**
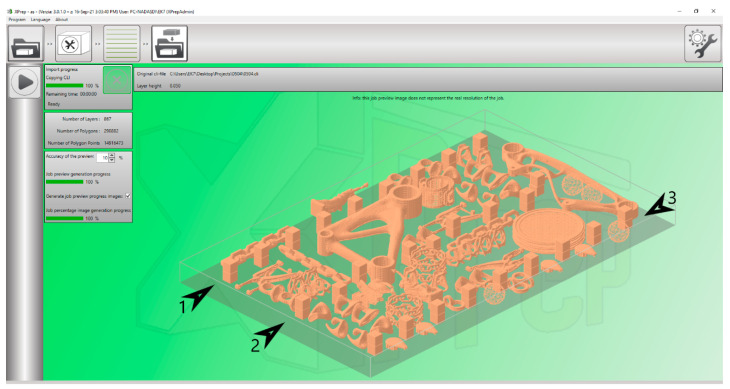
Distribution of cubes in the powder bed at the preprocessing stage.

From previous experiments and our experiment, shrinkage rates of *X*: 13%, *Y*: 13%, and *Z*: 16% were determined for further printing. Again, cubes were made to the size of the green part a = 20 mm. Unlike in the previous experiment, the placement of these samples in the powder bed will also be compared in this chapter, which may also affect the final deformation and accuracy. We will distinguish three hints according to the following Figure 14. From an economic point of view, there are also other parts in the powder bed to make printing as productive as possible. Arrows 1, 2, and 3 indicate the rows in which the dice are located. Optical measurement was performed on the same device—KEYENCE IM 8000.

**Figure 15:**
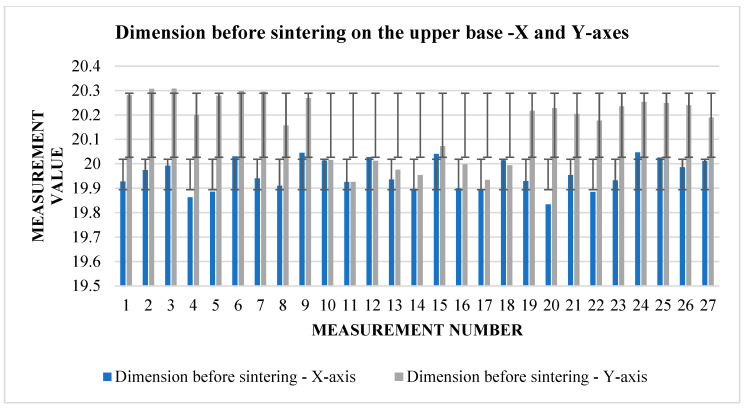
Graphical representation of Table 8—dimension before sintering—*X* and *Y*-axes (upper base).

**Figure 16:**
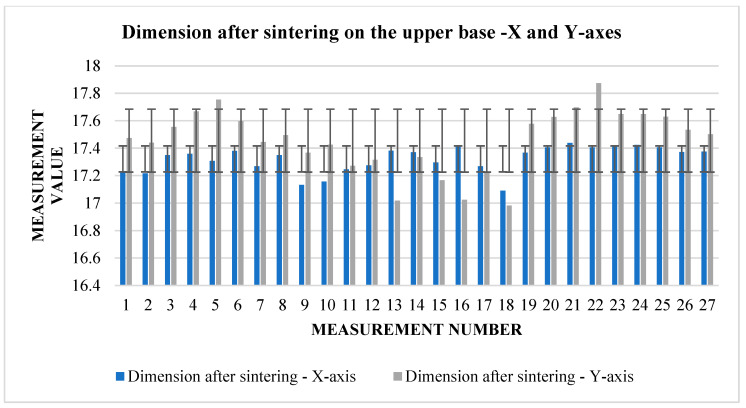
Graphical representation of Table 8—dimension after sintering—*X* and *Y*-axes (upper base).

From Table 8, we can read that the average shrinkage in the direction of the *X* and *Y* axes is slightly higher than the specified 13% in both. Therefore, a slight correction can be considered for further experiments so that the resulting dimensions are as close as possible to the requirements. Deviations between individual dimensions in the pre-sintering phase, but also after sintering, can be associated with printing parameters but also with the dedusting process, where fine layers can be removed when handling cubes. Fluctuations between dimensions can also be seen in the graphs (Figure 15 and Figure 16), showing shrinkage in both axes.

When comparing the values of shrinkage rates between cubes (Table 9), which were printed in different rows, it can be seen that in both directions on the upper base, the change in dimensions in the third row after sintering is always below 13%, although the average value of the dimensions after printing is not the lowest. The question is whether this difference originates from the printer or the layout of the cubes in the sintering furnace.

From Table 10, we can read that in the direction of the *X*-axis we are approaching the values we expected. The deviation is only 0.11%. In the *Y*-axis direction, this is almost 0.77%–which should be considered in further experiments.

**Figure 17:**
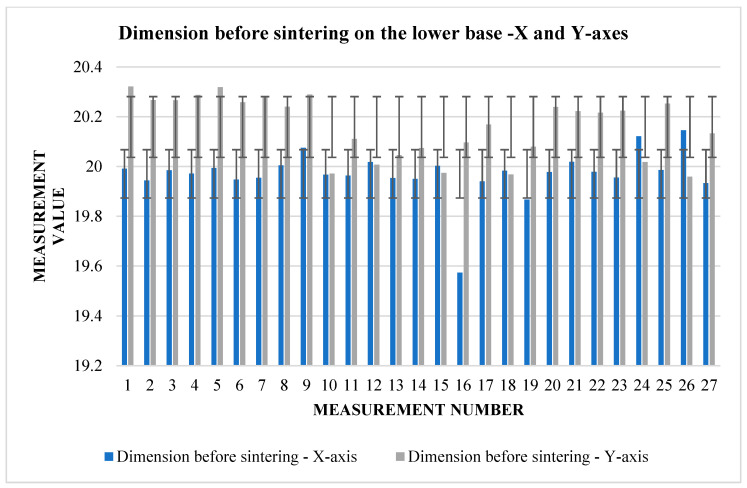
Graphical representation of Table 10—dimension before sintering—*X* and *Y*-axes (lower base).

**Figure 18:**
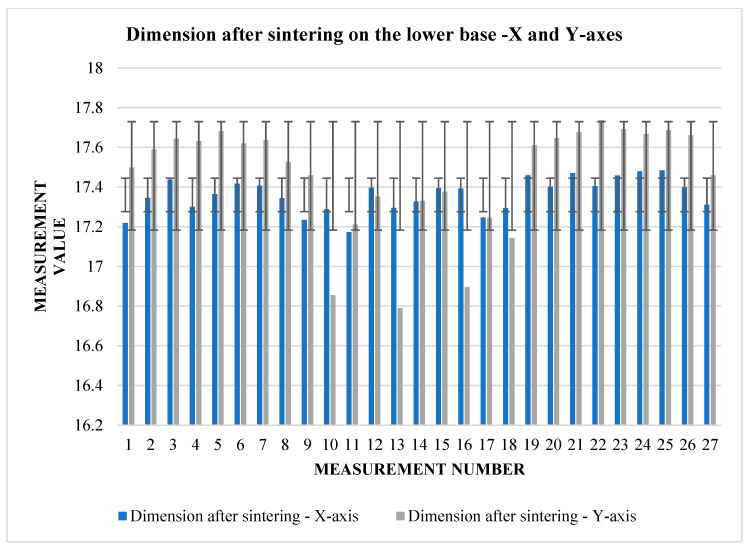
Graphical representation of Table 10—dimension after sintering—*X* and *Y*-axes (lower base).

**Figure 19:**
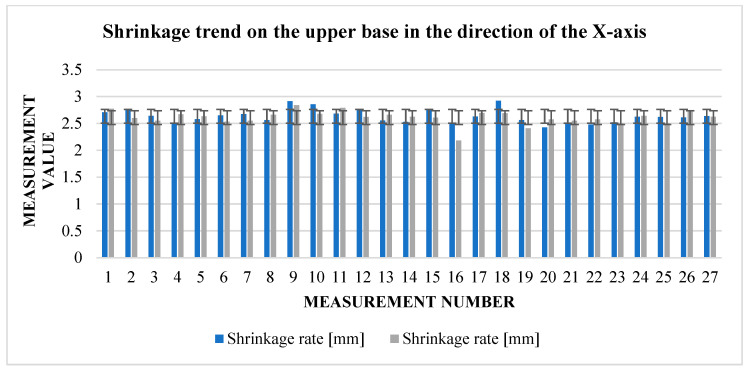
Graphical representation of Table 8 and Table 10—shrinkage in the direction of the *X*-axis.

**Figure 20:**
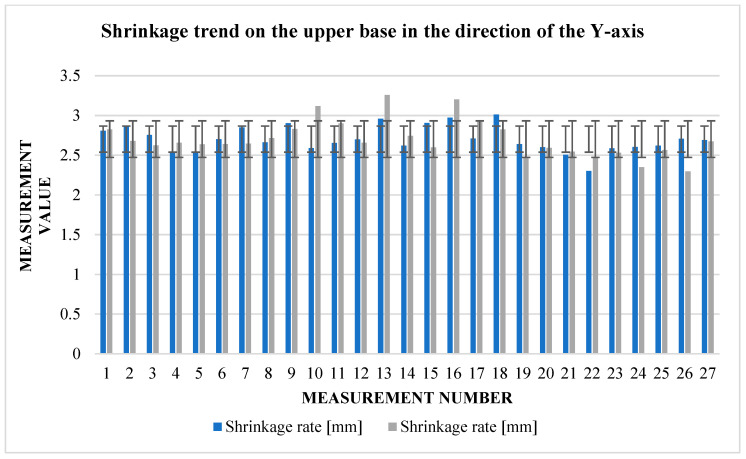
Graphical representation of Table 8 and Table 10—shrinkage in the direction of the *Y*-axis.

It can be seen from the graphs (Figure 17 and Figure 18) that the differences between dimensions are slightly stable in series 1 and 3 compared to the upper base. There are significant differences in the second row. When comparing lower base shrinkage in individual rows Table 11, an improvement can be seen in the third row compared to the upper base. However, the total shrinkage in the Y direction is constant (Figure 19 and Figure 20). This is due to rather high deviations in the second row.

In the direction of the *Z*-axis, the addition was set at 16%. The actual situation is 18.9%. Thus, the cubes were much more deformed than expected under the influence of gravity and other parameters, and the loss of dimensions could already be seen at a glance. It is, therefore, important to pay special attention to this direction in further attempts. The following graph shows the relative shrinkage values from Table 12.

**Figure 21:**
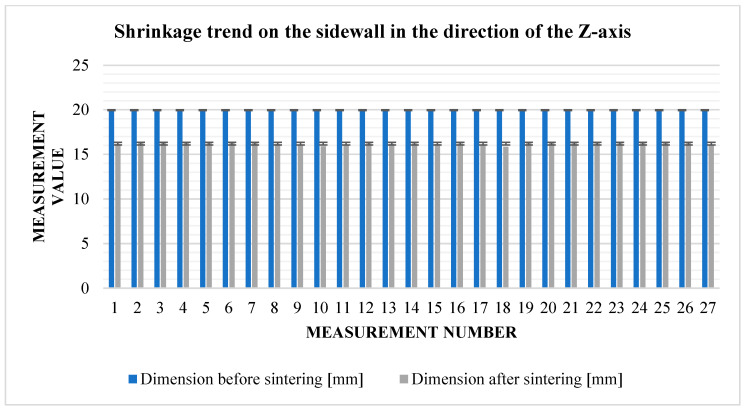
Graphical representation of Table 12—*Z*-axis shrinkage.

From Table 13, it can be seen that the greatest rate of shrinkage in the direction of the *Z*-axis (Figure 21) was in the second row. Overall, however, the figures in this regard are well above the planned 16%. This is due to the stacking printing method, where cubes printed at a lower level are affected by the weight of the cubes above them.

### 4.4. Hardness Tests

The hardness test was carried out according to Vickers (Table 14), carried out according to STN EN ISO (Slovak Technical Standard International European Standard Organization for Standardization) 6507-1 [43]. The punctures were made on a ground surface in the middle of the individual samples. The measured puncture diagonals are recalculated according to the device manufacturer’s tables. Hardness was measured on WPM HPO-250 (Leipzig, Germany).

In the material sheet from the manufacturer ExOne, the hardness according to Rockwell 67–71 HRB is indicated. After converting the units from our test, we obtain a value of 77.9 HRB. This means that we measured a hardness higher than the value stated by the manufacturer.

### 4.5. Static Tensile Test

**Figure 22:**
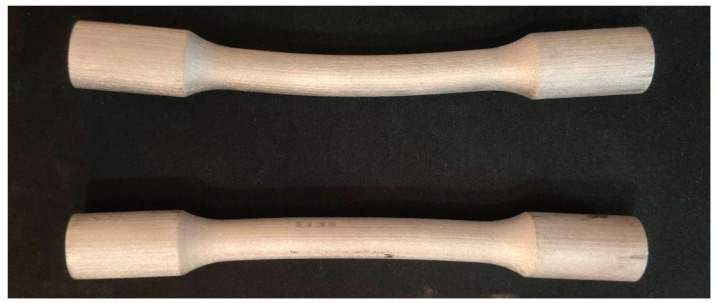
Deformed tensile samples.

Tensile tests were carried out according to STN EN (Slovak Technical Standard International European Standard) 10002-1. The proposed shape of the samples was circular in shape. The following Figure 22 shows deformed tensile samples.

**Figure 23:**
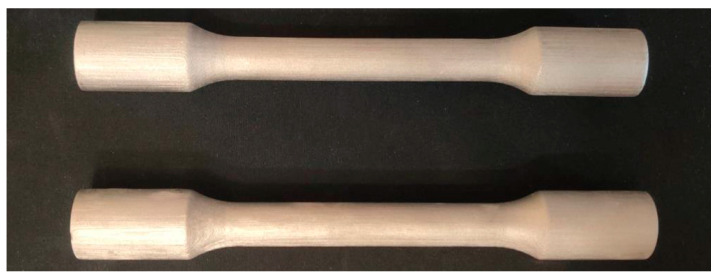
Samples printed using the Live Sinter simulation program.

The first printed samples were highly deformed not only in the axis of the samples but also in cross-section—it was not a circle but an ellipse. This deformation occurred during the sintering process. To print correction samples, a program was applied that predicts the rate of shrinkage and the direction of deformation during sintering. The result of the program is the design of such a geometry of the product, which is deformed into the desired shape during sintering. In addition to adjusting the geometry, the program (Figure 23) can also design supporting elements.

**Figure 24:**
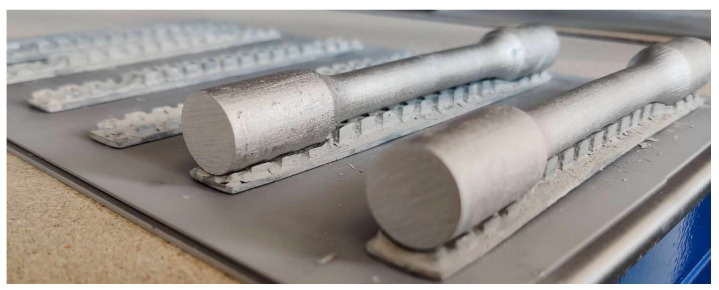
Samples with support elements suggested by Live Sinter.

The following Figure 24 shows samples with supporting elements suggested by Live Sinter.

**Figure 25:**
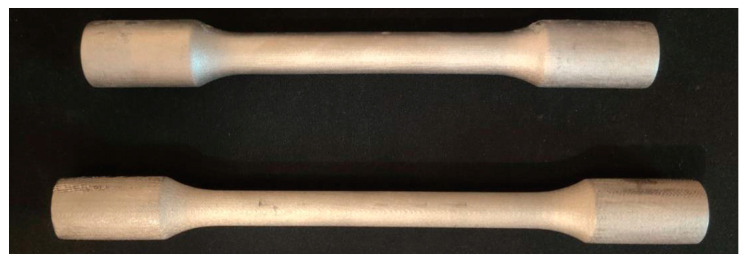
Samples after tensile test.

The first attempts in the tensile test (Figure 25) resulted repeatedly in the bars slipping out of the fixture. The material deformed above yield strength, but at values ≈ 455–460 MPa, it repeatedly slipped.

**Figure 26:**
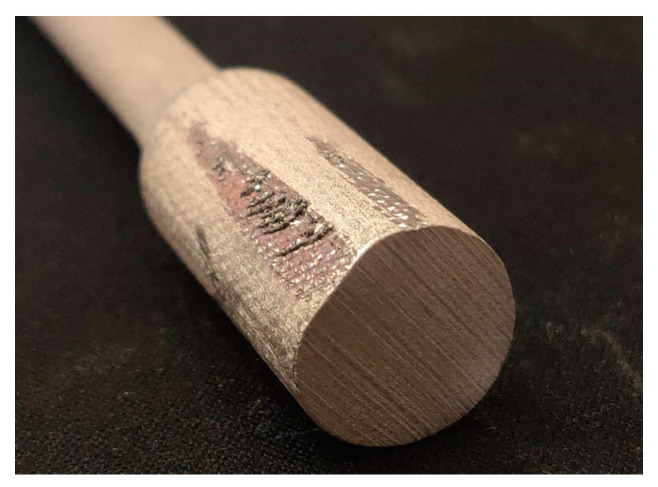
Traces of slipping out of the preparation.

The bottom sample in Figure 25. with considerable elongation and change in section thickness. However, slipping (Figure 26) from the fixture prevented it from breaking, and therefore, a different geometry will be chosen for further tests.

**Figure 27:**
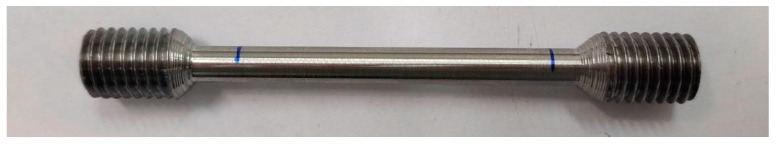
M12 threaded samples used in tensile test corrective experiments.

To eliminate the extraction of samples from the preparation, new rods were designed which were terminated with a thread (Figure 27). The samples were made by turning printed rods. These rods were then screwed into the special fixture in which the test was conducted.

**Figure 28:**
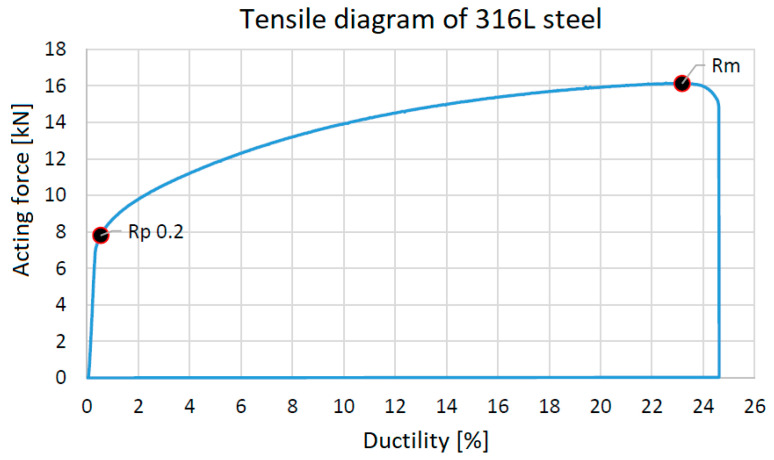
Tensile test conduct.

Turned samples were successfully tested. The tensile test results are shown in the diagram (Figure 28).

The testing used 10 samples and averaged the results. The following Table 15 compares the values measured by tests and the material sheet supplied with ExOne’s 316L steel.

**Figure 29:**
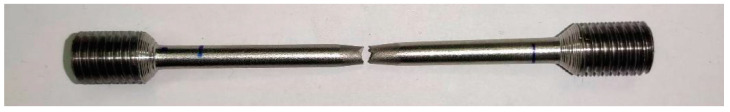
Sample after rupture.

When comparing the results, we can say that the characteristics declared by the manufacturer agree with the values obtained by the experiment (Figure 29). The test samples were pushed to plane *XY*. The current state of optimization of the printer does not yet allow printing a similar sample in the vertical direction.

### 4.6. Spectrometry-Verification of Chemical Composition

**Figure 30:**
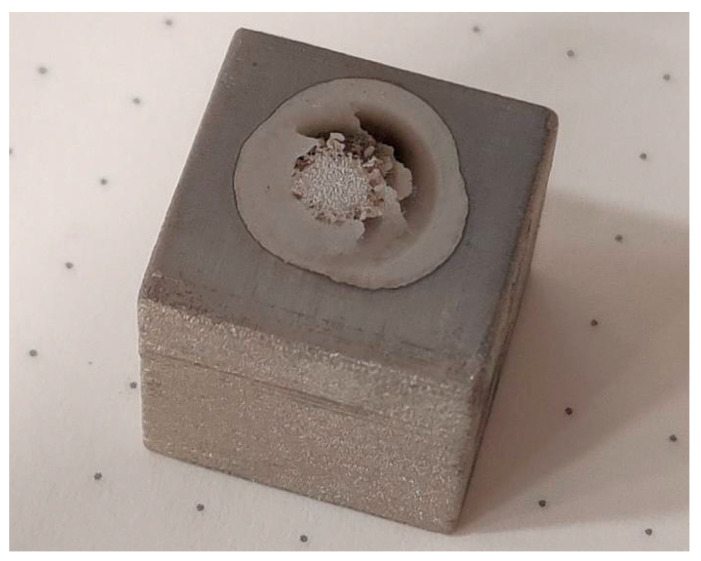
The surface of the sample after discussing the surface.

Verification of the chemical composition of the samples was performed on the BELEC COMPACT PORT spectrometer. The principle of chemical analysis consists of discriminating the surface of the sample with an argon gun. The following Figure 30 shows the sample after the test.

Spectrometry determined the content not only of the elements declared by the manufacturer but also of others, such as P, S, Cu, and Nb (Table 16). The result of spectrometry showed that the powdered material has values that fall into the material sheet from the manufacturer ExOne. The only element that is outside the interval is carbon (Table 17). Based on the chemical composition, the spectrometer estimated steel grade 1.4401, marked X5CrNiMo17-12-2, in US (United States) standard (AISI (American Iron and Steel Institute)) designated 316. Stainless austenitic chromo-nickel-molybdenum steel has the same properties as 316L steel but is not resistant to intergranular corrosion.

### 4.7. Density

An important parameter that has a significant impact on the quality and mechanical properties of the printed components is density. The manufacturer ExOne indicates a density in the range of 7.6–7.9 g/cm^3^ for 316L. In our experiment, we will measure the relative density of cubes before and after sintering, with an indicator of 7.97 g/cm^3^. The measured samples were randomly selected from the first, second, and third series of the desktop of the X160Pro printer.

From Table 18. we can see that the cubes entered the process with an input relative density ≈ 61.21%. The highest values were achieved in the third row. Further research could include a deeper comparison of the influence of sample placement on the resulting density.

**Figure 31:**
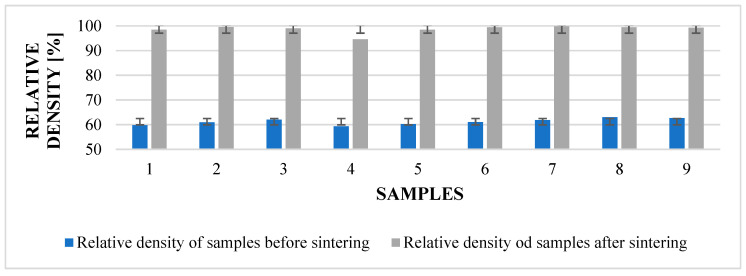
Comparative graph between the densities obtained before and after sintering.

Table 19 shows the effect of shrinkage on the finite relative density. The resulting relative density is 98.7% (Figure 31). Again, we can see that in the three series, the values are the highest. The value achieved by us is in accordance with the value declared by the manufacturer ExOne in the material sheet for steel 316L.

### 4.8. Macrostructure of Steel 316L

The microstructure of the sample made of 316L steel was assessed on the KEYENCE VH-Z100R digital microscope.

**Figure 32:**
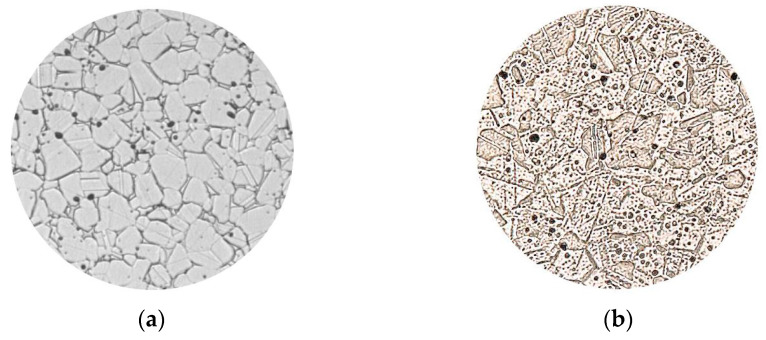
Samples: (**a**) comparison of the microstructure given in the material specification [6], (**b**) microstructure captured on a digital microscope. Scale bar: 1:500.

Figure 32 shows a comparison between the microstructure given in the material specification and the microstructure captured on a digital microscope.

We can say that with more thorough polishing of the sample, the resulting microstructure would be closer to the one stated by the manufacturer ExOne. Our sample shows a slightly higher degree of porosity. However, it was not significantly reflected in the resulting density and mechanical properties.

### 4.9. Cost-Effective Binder Jetting Technology

For an approximate estimate of the economic advantage of binder jetting technology, we compared the production of a six-edge screw M12 x 100 made of stainless steel, similar in properties to 316L steel. The calculation was carried out using an online calculator offered by the manufacturer ExOne [42]. In the application, it is necessary to enter the type of machine on which the printing will take place, the material of the powder, the dimensions of the final component, its approximate volume in mm^3^, and the method of distribution or percentage of filling the powder bed. The output values include approximate prices for powder, binder, and detergents.

The resulting values calculated by the calculator are shown in Table 20.

The average price of a finished M12 x 100 screw, made using conventional technology, is approximately 1.48 € [44]. It is necessary to take into account the fact that, at the moment, we cannot produce a screw of the required precision and quality using binder jetting technology without finishing modifications of turning, grinding, or polishing. These would significantly increase the resulting cost of one component. It is also important to mention current electricity prices. The most energy-intensive process is the sintering phase in a sintering furnace, consuming approximately 1.5 MWh per sintering cycle.

**Figure 33:**
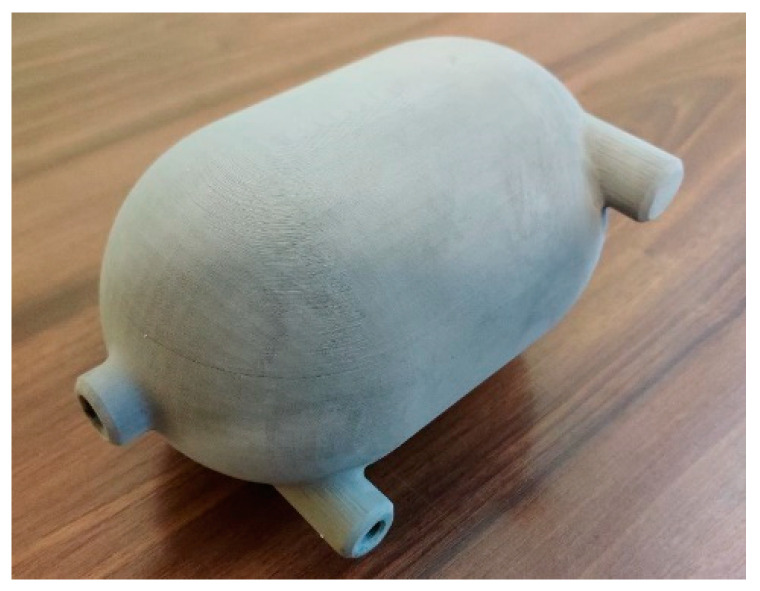
Hydrogen tank made by binder jetting technology.

Based on these facts, we can say that finding a product suitable for series production for 316L steel is difficult. A more suitable application of binder jetting technology is piece and prototype production. It is especially suitable for components where we can take advantage of the fact that the component is printed as a whole and thus eliminate the need to use a combination of conventional technologies (e.g., bending and welding), which would shorten production time and reduce production costs. One of the proposed parts for the use of binder jetting technology is a pressurized hydrogen tank (Figure 33) with special cooling channels.

By using binder jetting technology, several technological operations are eliminated. The inside of the tank contains special channels that were made during one operation together with the tank itself. This eliminates the technology needed to make channels and connect them to the tank. The tank itself is also printed as a single unit without the need for welding. By producing the tank and its interior as a whole, the unsaturated powder is enclosed in the cavity of the tank. Therefore, it is necessary to remove it with compressed air or other technology that will not damage the geometry of the component before sintering.

**Figure 34:**
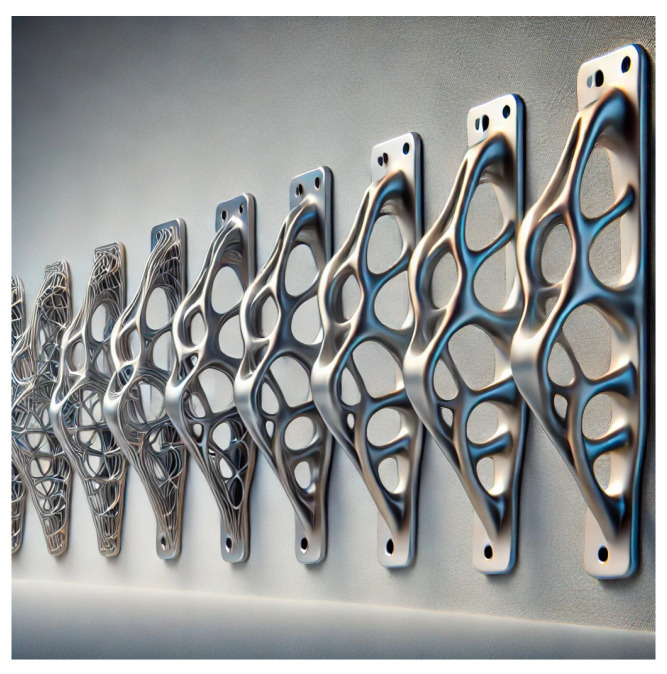
Gradual removal of material from the structural element by topological optimization.

**Figure 35:**
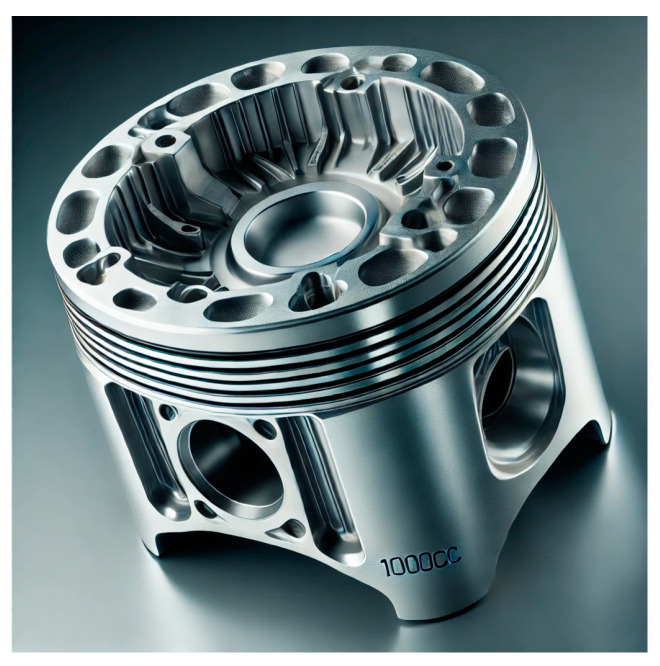
Piston made by 3D printing technology.

In addition to minimizing the technologies required to produce certain components, the great benefit of additive binder jetting is the reduction in the weight of parts. The fact that the technology theoretically does not know an obstacle in terms of the manufacturability of geometrically complex parts opens up space for the use of so-called topological optimization and generative design. The principle of topological optimization (Figure 34) is based on the design of a structurally and geometrically ideal shape of a component by gradual material removal (Figure 35). Generative design, on the other hand, designs a geometric shape by gradually adding material. Suitable software generates a part based on boundary conditions such as material, load, and degrees of freedom that would often not be possible to produce with conventional technologies or would be economically disadvantageous. The final geometry of the product is as lightweight as possible while meeting the strength and mechanical requirements. The minimum weight of the currently ever-increasing fuel prices is particularly suitable in the automotive industry. In addition to the automotive industry, it is also used in the construction, medical, and aerospace industries.

The piston (Figure 35) was designed by Porsche using topological optimization. Additive technology was used to produce it, reducing weight by up to 10% on such a small component [45].

## 5. Discussion

Binder jetting technology has enormous potential, and current trends indicate its growing importance in industrial applications. It now enables the printing of the largest metal parts, which puts it at the forefront among other additive technologies. It is extremely suitable for the automotive, marine, aerospace, and even aerospace industries, requiring minimal finishing technological modifications, known as post-processing.

The advantages of this technology lie in its ability to create complex geometries that would be unfeasible or costly with conventional manufacturing methods. In addition, binder jetting allows the use of a wide range of materials, which opens the door to innovative applications and new business opportunities.

However, there is much room for improvement in this technology, not only in terms of hardware but also in terms of software. Improving data pre-preparation for devices without design interventions is a key aspect that can significantly impact print quality and efficiency. The authors of the article have two devices with binder jetting technology at their disposal and plan to optimize them in the future based on data preparation. This step is extremely important, as optimizing the data to eliminate material density depending on the location of the print area can lead to higher material homogeneity. Homogeneity is very important for the mechanical properties and reliability of the final products.

The expansion of binder jetting technology and the acceleration of its deployment can lead to a reduction in production costs, which will increase its competitiveness against other additive technologies as well as traditional production methods. In addition, advances in software tools for data preparation can bring better control over the printing process, allowing for even higher product quality and accuracy.

Another important aspect to consider is the environmental impact of binder jetting technology. This technology has the potential to reduce waste and material consumption compared to traditional production methods, which is in line with the global pressure for more sustainable production processes. In addition, reducing costs and increasing efficiency can lead to wider adoption of this technology, which could have a positive impact on the entire industrial ecosystem.

As experiments with the 316L material have shown, achieving the specified product properties implies the perfect alignment of several stages of the production process. The most important influence on the final accuracy of the product is the sintering process in the sintering furnace. In this step, the inaccuracies achieved at the powder and binder layering stage are multiplied as a result of shrinkage. The resulting geometry is also significantly influenced by the powder’s own weight. This was also confirmed by optical measurement of samples, where the vertical direction appeared to be the most problematic direction of printing. In this axis, shrinkage reached its highest values. In addition to dimensional accuracy, compliance with geometric tolerances is also a challenge. A simulation program predicting deformation offers a potential solution to deviations that occur during sintering. A deeper investigation of the parameters and their effects in the printing, dedusting, and sintering phases is a must.

Although binder jetting technology already offers significant advantages today, its future lies in continuous development and optimization. Continued investment in research and development, collaboration between academia and industry, and a focus on improving software and hardware solutions are key to unlocking the full potential of this technology. This not only improves the quality and efficiency of production but also makes the industrial process more sustainable and economically advantageous.

The economic advantage of technology depends on several factors. The most important factor is the manufacturability of another technology. Another important factor is the material, which predetermines the field of application. The 316L steel we examine is the most widely used material in PM. Its practical use in terms of economic return is inefficient when compared to the mass production of conventionally produced components. A negative factor affecting economic profitability is the production time and the sintering itself. The sintering process is both an energy-consuming and time-consuming process that negatively affects economic returns. An unmistakable factor is that binder jetting is currently unable to produce parts without the need for finishing operations. The production flexibility of binder jetting is particularly suitable for piece or prototype production. To achieve a precisely defined geometry, it is necessary to use the addition of material for finishing operations, which increases production costs. The production of similarly complex parts requires considerable research into the effects of process parameters that directly affect the final density, accuracy, and properties of the final product.

## 6. Conclusions

The additive approach of binder jetting technology, coupled with the wide range of materials available, is a potential solution even for conventionally non-manufacturing components.

The optimal production setup has a significant impact on the overall economy of the process binder jetting technology. The influence of parameters such as powder layering rate, binder deposition, powder and binder thickness, sintering time and sintering temperatures, automation of dedusting processes, parts handling, and others are other areas of research whose benefits for binder jetting will open up new possibilities of application.

The measurement found that the shape in the vertical direction is significantly influenced by gravity, which deforms the printed parts differently depending on the vertical location in the printing area. The difference between the printed samples in the upper and lower base in the vertical direction was 3%. During the data design, it must be calculated according to the location of the printed part.

The properties of the material declared by the manufacturer have been verified by static tensile test and hardness test. With a relative density of almost 99%, the resulting sample properties were comparable to conventionally treated 316L stainless steel.

The results of the tensile test proved that the material is able to withstand a stress of more than 530 MPa.

The hardness of the structure was above the manufacturer’s declared value—77.9 HRB. At a relative density above 99% or using infiltration, these values would be even higher. The chemical composition of metallic powder has been verified by spectrometry. When observing the microstructure with a digital microscope, porosity could be seen, which, however, did not affect the resulting material properties too negatively.

Sintering in a sintering furnace is one of the most expensive items from the cost of making binder jetting. One cycle of sintering in a sintering furnace consumes 1.6 MWh of electricity, which, at current astronomical prices in Europe, makes it impossible to use this technology for commercial use. At a price of €0.50 per 1kWh of electricity without VAT, only the cost of sintering is €800; with a price of powder of €12 per kilogram, the cost of electricity is higher than 65 kg of material. It is, therefore, necessary to use the maximum capacity of the sintering furnace for efficiency because the price of the consumed electricity is distributed over a larger amount of sintered material. As it has emerged from measurements, when the powder itself is heavily loaded on the lower part of the printing base, there is a more significant deformation and different deformation compared to the parts printed in the upper part.

The authors are currently working on optimizing the data for printing-better determining deformations in the vertical direction, which were also reflected in the measurements already presented. Another research is the mechanical improvement of the application parts of the device, which is already the subject of a patent procedure.

## Figures and Tables

**Table 1:** Currently used metallic powder materials [29].

Base Material	Colloquial/Commercial Name	Alloy Marking (According to American Standard ASME *)
Iron	Stainless steels	316, 316L, 304, 410, 420
	Maraging steel	18 MAR 300
	Hardening tool steel	15-5
	Hardening stainless steel	17-4PH, 13-8
	Tool steels	H13, S7
Nickel	Superalloys	IN-718, IN-625, Waspalloy, René 41
Cobalt	Superalloys	Co-Cr-Mo, Satellite class 6, and class 21
Titanium	Ti-rich	Ti-6Al-4V, Ti-6Al-7Nb, Ti-6Al-2Sn-4Zr-2Mo
	Commercially used pure Ti	CP
Copper	Bronze	Cu-Sn
Aluminum	Alloys for casting	Al-Si-10Mg, Al4047, Al-Si-7Mg-Cu, Al-12Si

Explanation: * American Society of Mechanical Engineers.

**Table 2:** Material properties declared by the manufacturer ExOne [41].

Material Property	Test Method	Values
Ultimate strength Rm	ASTM E8	X, Y: 450–580 MPa Z: 450–520 MPa
Yield strength (0.2%)		X, Y: 140–220 MPa Z: 140–220 MPa
Extension to infringement		X, Y: 40–55% from 40–50%
Modulus of elasticity		X, Y: 190–220 GPa W: 180–190 GPa
Hardness	ASTM E18	67–71 HRB *
Impact toughness	ASTM E23	55–75 J
Poisson number		0.28–0.30
Relative density		96–99%
Density		7600–7900 kg·m^−2^
Surface roughness		Ra 3–12 μm

Explanation: * Rockwell Hardness Test on the B Scale.

**Table 3:** Chemical composition of steel material 316L [41].

Chemical Composition	
Element	[%]
Nickel	10–14
Chromium	16–18
Carbon	max. 0.03
Molybdenum	2–3
Manganese	max. 2.0
Silicon	max. 1.0
Iron	remainder

**Table 4:** Parameters of printing and powder used.

Device	ExOne DesktopMetal X160Pro^®^
Powder layer height	50 μm
The speed of movement of the recoater	100 mm/s
Roller speed (opposite)	800 rpm
Material	Steel 316L
PSD	
D10	3–4 μm
D50	11–12 μm
D90	21–22 μm
Particle shape	Spherical powder (balls)
	Produced by gas atomization

**Table 5:** Comparison of dimensional changes before and after sintering in the direction of the *X* and *Y* axes on the upper base.

Measured Wall					Upper Base			
Measurement Orientation								
*X*					*Y*			
Dimension before Sintering [mm]		Dimension after Sintering [mm]	Shrinkage Rate		Dimension before Sintering [mm]	Dimension after Sintering [mm]	Shrinkage Rate	
			[mm]	[%]			[mm]	[%]
20.007		17.591	2.416	12.08	20.334	17.763	2.571	12.64
19.923		17.585	2.338	11.74	20.381	17.828	2.553	12.53
20.071		17.547	2.524	12.58	20.331	17.877	2.454	12.07
20.001		17.544	2.457	12.28	20.347	17.804	2.543	12.50
19.983		17.579	2.404	12.03	20.238	17.856	2.382	11.77
20.076		17.608	2.468	12.29	20.254	17.806	2.448	12.09
20.010		17.518	2.492	12.45	20.067	17.788	2.279	11.36
19.914		17.636	2.278	11.44	19.902	17.685	2.217	11.14
20.066		17.662	2.404	11.98	20.113	17.838	2.275	11.31
20.030		17.489	2.541	12.69	20.154	17.797	2.357	11.69
20.033		17.613	2.420	12.08	20.268	17.785	2.483	12.25
20.006		17.617	2.389	11.94	20.304	17.882	2.422	11.93
19.998		17.654	2.344	11.72	19.993	17.868	2.125	10.63
20.038		17.577	2.461	12.28	20.269	17.777	2.492	12.29
20.052		17.552	2.500	12.47	20.331	17.728	2.603	12.80
19.987		17.667	2.320	11.61	20.105	17.682	2.423	12.05
20.039		17.598	2.441	12.18	20.289	17.791	2.498	12.31
20.002		17.611	2.391	11.95	20.381	17.833	2.548	12.50
20.051		17.651	2.400	11.97	20.305	17.814	2.491	12.27
20.062		17.597	2.465	12.29	20.274	17.782	2.492	12.29
Min.	19.914	17.489	2.278	11.44	19.902	17.682	2.125	10.63
Max.	20.076	17.667	2.541	12.69	20.381	17.882	2.603	12.80
AP	20.017	17.595	2.423	12.10	20.232	17.799	2.433	12.02
SD	0.04311	0.04681			0.12961	0.0543		

**Table 6:** Comparison of dimensional changes before and after sintering in the direction of the *X* and *Y* axes on the lower base.

Measured Wall					Lower Base			
Measurement Orientation								
*X*					*Y*			
Dimension before Sintering [mm]		Dimension after Sintering [mm]	Shrinkage Rate		Dimension before Sintering [mm]	Dimension after Sintering [mm]	Shrinkage Rate	
			[mm]	[%]			[mm]	[%]
19.976		17.571	2.405	12.04	20.238	17.809	2.429	12.00
19.916		17.491	2.425	12.18	20.278	17.826	2.452	12.09
19.989		17.473	2.516	12.59	20.268	17.814	2.454	12.11
19.937		17.497	2.440	12.24	20.232	17.833	2.399	11.86
19.961		17.585	2.376	11.90	20.810	17.884	2.926	14.06
20.204		17.553	2.651	13.12	20.008	17.77	2.238	11.19
19.942		17.425	2.517	12.62	20.006	17.755	2.251	11.25
19.879		17.587	2.292	11.53	20.053	17.621	2.432	12.13
19.983		17.593	2.390	11.96	20.166	17.812	2.354	11.67
19.891		17.480	2.411	12.12	20.088	17.807	2.281	11.36
19.945		17.558	2.387	11.97	20.265	17.791	2.474	12.21
19.933		17.634	2.299	11.53	20.135	17.781	2.354	11.69
19.912		17.545	2.367	11.89	20.009	17.772	2.237	11.18
19.978		17.574	2.404	12.03	20.091	17.766	2.325	11.57
19.926		17.512	2.414	12.11	20.112	17.694	2.418	12.02
20.012		17.650	2.362	11.80	20.233	17.676	2.557	12.64
19.921		17.411	2.510	12.60	20.198	17.813	2.385	11.81
19.993		17.513	2.480	12.40	20.214	17.762	2.452	12.13
19.917		17.539	2.378	11.94	20.017	17.669	2.348	11.73
19.946		17.501	2.445	12.26	20.195	17.715	2.48	12.28
Min.	19.879	17.411	2.292	11.53	20.006	17.621	2.237	11.18
Max.	20.204	17.65	2.651	13.12	20.810	17.833	2.557	12.64
AP	19.958	17.535	2.423	12.14	20.148	17.762	2.385	11.84
SD	0.06607	0.06135			0.17133	0.06301		

**Table 7:** Comparison of dimension changes before and after sintering in the direction of the *Z*-axis.

Measured Wall			Side Wall	
Measurement Orientation				
From				
Dimension before Sintering [mm]		Dimension after Sintering [mm]	Shrinkage Rate	
			[mm]	[%]
20.009		16.865	3.144	15.71
20.006		16.767	3.239	16.19
20.037		16.788	3.249	16.22
20.029		16.795	3.234	16.15
19.806		16.801	3.005	15.17
20.04		16.849	3.191	15.92
19.985		16.73	3.255	16.29
19.859		16.784	3.075	15.48
20.012		16.920	3.092	15.45
20.003		16.789	3.214	16.07
19.997		16.793	3.204	16.02
19.985		16.912	3.073	15.38
20.032		16.796	3.236	16.15
19.895		16.771	3.124	15.70
19.982		16.714	3.268	16.35
20.011		16.906	3.105	15.52
19.881		16.871	3.010	15.14
19.933		16.836	3.097	15.54
20.007		16.793	3.214	16.06
20.012		16.811	3.201	16.00
Min.	19.806	16.714	3.005	15.14
Max.	20.040	16.92	3.268	16.35
AP	19.976	16.815	3.162	15.83
SD	0.064	0.05564		

**Table 8:** Comparison of dimensional changes before and after sintering in the direction of the *X* and *Y* axes on the upper base.

Measured Wall					Upper Base				
Measurement Orientation									
*X*					*Y*				
Dimension before Sintering [mm]		Dimension after Sintering [mm]	Shrinkage Rate		Dimension before Sintering [mm]	Dimension after Sintering [mm]	Shrinkage Rate		
			[mm]	[%]			[mm]	[%]	
19.927		17.222	2.705	13.57	20.281	17.473	2.808	13.85	1st series
19.974		17.217	2.757	13.80	20.307	17.441	2.866	14.11	
19.992		17.35	2.642	13.22	20.308	17.554	2.754	13.56	
19.863		17.36	2.503	12.60	20.201	17.668	2.533	12.54	
19.886		17.308	2.578	12.96	20.279	17.754	2.525	12.45	
20.030		17.38	2.650	13.23	20.298	17.596	2.702	13.31	
19.940		17.268	2.672	13.40	20.295	17.446	2.849	14.04	
19.910		17.349	2.561	12.86	20.157	17.494	2.663	13.21	
20.045		17.133	2.912	14.53	20.269	17.368	2.901	14.31	
20.013		17.157	2.856	14.27	20.016	17.425	2.591	12.94	2nd series
19.926		17.246	2.680	13.45	19.926	17.272	2.654	13.32	
20.028		17.275	2.753	13.75	20.012	17.315	2.697	13.48	
19.936		17.382	2.554	12.81	19.976	17.018	2.958	14.81	
19.897		17.370	2.527	12.70	19.954	17.335	2.619	13.13	
20.040		17.296	2.744	13.69	20.072	17.167	2.905	14.47	
19.899		17.411	2.488	12.50	19.998	17.025	2.973	14.87	
19.896		17.268	2.628	13.21	19.934	17.226	2.708	13.58	
20.014		17.091	2.923	14.60	19.994	16.982	3.012	15.06	
19.929		17.367	2.562	12.86	20.217	17.577	2.64	13.06	3rd series
19.834		17.407	2.427	12.24	20.228	17.627	2.601	12.86	
19.954		17.439	2.515	12.60	20.204	17.697	2.507	12.41	
19.885		17.406	2.479	12.47	20.177	17.874	2.303	11.41	
19.932		17.413	2.519	12.64	20.235	17.648	2.587	12.78	
20.047		17.424	2.623	13.08	20.253	17.649	2.604	12.86	
20.026		17.407	2.619	13.08	20.249	17.629	2.62	12.94	
19.986		17.373	2.613	13.07	20.24	17.533	2.707	13.37	
20.011		17.376	2.635	13.17	20.19	17.501	2.689	13.32	
Min.	19.834	17.091	2.427	12.24	19.926	16.982	2.525	12.45	
Max.	20.045	17.411	2.923	14.60	20.308	17.754	3.012	15.06	
AP	19.949	17.293	2.656	13.31	20.136	17.388	2.748	13.65	
SD	0.06104	0.09301			0.12895	0.22521			

**Table 9:** The difference between shrinkage in different rows.

Shrinkage X [%]		Shrinkage Y [%]	
1st series	13.35	1st series	13.49
2nd series	13.44	2nd series	13.96
3rd series	12.80	3rd series	12.78

**Table 10:** Comparison of dimensional changes before and after sintering in the direction of the *X* and *Y* axes on the lower base.

Measured Wall					Lower Base				
Measurement Orientation									
*X*					*Y*				
Dimension before Sintering [mm]		Dimension after Sintering [mm]	Shrinkage Rate		Dimension before Sintering [mm]	Dimension after Sintering [mm]	Shrinkage Rate		
			[mm]	[%]			[mm]	[%]	
19.991		17.219	2.772	13.87	20.321	17.498	2.823	13.89	1st series
19.944		17.345	2.599	13.03	20.267	17.589	2.678	13.21	
19.985		17.437	2.548	12.75	20.266	17.644	2.622	12.94	
19.972		17.301	2.671	13.37	20.287	17.632	2.655	13.09	
19.994		17.364	2.630	13.15	20.318	17.682	2.636	12.97	
19.948		17.417	2.531	12.69	20.258	17.62	2.638	13.02	
19.955		17.407	2.548	12.77	20.281	17.637	2.644	13.04	
20.005		17.344	2.661	13.30	20.24	17.526	2.714	13.41	
20.075		17.235	2.840	14.15	20.289	17.459	2.83	13.95	
19.967		17.288	2.679	13.42	19.972	16.855	3.117	15.61	2nd series
19.964		17.173	2.791	13.98	20.111	17.211	2.9	14.42	
20.018		17.397	2.621	13.09	20.007	17.352	2.655	13.27	
19.954		17.295	2.659	13.33	20.046	16.79	3.256	16.24	
19.95		17.327	2.623	13.15	20.074	17.331	2.7425	13.66	
20.003		17.394	2.609	13.04	19.974	17.376	2.598	13.01	
19.574		17.392	2.182	11.15	20.097	16.896	3.201	15.93	
19.94		17.247	2.693	13.51	20.168	17.244	2.924	14.50	
19.983		17.294	2.689	13.46	19.968	17.143	2.825	14.50	
19.867		17.459	2.408	12.12	20.08	17.61	2.47	12.30	3rd series
19.978		17.402	2.576	12.89	20.239	17.647	2.592	12.81	
20.019		17.47	2.549	12.73	20.222	17.676	2.546	12.59	
19.979		17.404	2.575	12.89	20.216	17.737	2.479	12.26	
19.956		17.458	2.498	12.52	20.224	17.692	2.532	12.52	
20.121		17.479	2.642	13.13	20.018	17.668	2.35	11.74	
19.986		17.484	2.502	12.52	20.252	17.687	2.565	12.67	
20.145		17.399	2.746	13.63	19.959	17.661	2.298	11.51	
19.933		17.31	2.623	13.16	20.133	17.459	2.674	13.28	
Min.	19.574	17.173	2.182	11.15	19.968	16.790	2.470	12.30	
Max.	20.075	17.459	2.84	14.15	20.321	17.682	3.256	16.24	
AP	19.953	17.337	2.617	13.11	20.163	17.387	2.776	13.77	
SD	0.09531	0.08295			0.11979	0.26799			

**Table 11:** The difference between shrinkage in different rows.

Shrinkage X [%]		Shrinkage Y [%]	
1st series	13.23	1st series	13.28
2nd series	13.12	2nd series	14.53
3rd series	12.84	3rd series	12.41

**Table 12:** Comparison of dimension changes before and after sintering in the direction of the *Z*-axis.

Measuring Wall		Side Wall			
Measurement Orientation					
From					
Dimension before Sintering [mm]		Dimension after Sintering [mm]	Shrinkage Rate		
			[mm]	[%]	
20.046		16.186	3.860	19.26	1st series
19.975		16.258	3.717	18.61	
19.971		16.409	3.562	17.84	
20.012		16.338	3.674	18.36	
20.019		16.44	3.579	17.88	
19.97		16.421	3.549	17.77	
19.948		16.282	3.666	18.38	
20.03		16.179	3.851	19.23	
20.05		16.069	3.981	19.86	
19.953		15.914	4.039	20.24	2nd series
19.991		16.046	3.945	19.73	
19.944		16.260	3.684	18.47	
19.961		16.183	3.778	18.93	
20.002		16.223	3.779	18.89	
19.957		16.147	3.810	19.09	
20.02		16.230	3.790	18.93	
19.946		16.000	3.946	19.78	
19.927		15.868	4.059	20.37	
19.837		16.246	3.591	18.10	3rd series
19.9		16.266	3.634	18.26	
20.007		16.274	3.733	18.66	
19.886		16.307	3.579	18.00	
19.964		16.339	3.625	18.16	
19.984		16.335	3.649	18.26	
19.991		16.279	3.712	18.57	
19.911		16.256	3.655	18.36	
20.005		16.002	4.003	20.01	
Min.	19.837	15.868	3.549	17.77	
Max.	20.050	16.44	4.059	20.37	
AP	19.982	16.198	3.775	18.90	
SD	0.04885	0.14475			

**Table 13:** The difference between shrinkage in different rows.

Shrinkage from [%]	
1st series	18.57
2nd series	19.38
3rd series	18.49

**Table 14:** Vickers hardness test values.

	u1 [mm]	u2 [mm]	Diameter [mm]	HV10	HRB
1. Measurement	0.350	0.360	0.3550	147	-
2. Measurement	0.360	0.349	0.3545	148	-
3. Measurement	0.351	0.350	0.3505	151	-
4. Measurement	0.350	0.350	0.3500	151	-
5. Measurement	0.358	0.352	0.3550	147	-
Diameter				148.8	77.9

**Table 15:** Comparison of values obtained by experiment and material sheet.

	Manufacturer’s Declared Value	The Value Obtained by the Experiment
Rm [MPa]	450–580	530–545
Rp 0.2 [MPa]	140–220	245–259
A * [%]	40–50	39.5–41

Explanation: * Elongation at break.

**Table 16:** Spectrometry measurement results.

Measurement	Fe [%]	C [%]	Si [%]	Mn [%]	P [%]	S [%]	Cu [%]	
1	67.760	0.052	0.870	1.310	0.016	<0.002	0.231	
2	67.670	0.044	0.924	1.352	0.017	<0.002	0.225	
3	67.580	0.040	0.924	1.357	0.017	<0.002	0.224	
SD	0.07348	0.00498	0.02254	0.02107	0.00047	0	0.00309	
**Measurement**	**Al** **[%]**	**Cr** **[%]**	**Mo** **[%]**	**Ni** **[%]**	**V** **[%]**	**Ti** **[%]**	**Nb** **[%]**	**Co** **[%]**
1	0.024	17.030	2.232	10.280	0.059	0.005	0.017	0.108
2	0.022	16.750	2.173	10.580	0.076	0.006	0.032	0.122
3	0.022	16.700	2.142	10.720	0.082	0.006	0.037	0.125
SD	0.00094	0.14522	0.03733	0.18354	0.00974	0.00047	0.00849	0.00740

**Table 17:** Comparison of spectrometry results and material sheet.

	Fe [%]	Ni [%]	Cr [%]	C [%]	Mo [%]	Mn [%]	Si [%]
Diameter	67.670	10.527	16.827	0.045	2.182	1.340	0.906
ExOne	remainder	10–14	16–18	max. 0.03	2–3	max. 2.0	max. 1.0

**Table 18:** Relative density of samples before sintering.

		X [mm]	Y [mm]	Z [mm]	V [mm^3^]	Weight [g]	Density [g/cm^3^]	Relative Density [%]
Series 1	Sample 1	19.927	20.281	20.046	8101.380	38.254	4.72	59.77
	Sample 2	19.974	20.307	19.975	8102.100	38.994	4.81	60.92
	Sample 3	19.992	20.308	19.971	8108.177	39.761	4.90	62.07
Series 2	Sample 4	20.013	20.016	19.953	7992.777	37.454	4.69	59.32
	Sample 5	19.926	19.926	19.991	7937.336	37.781	4.76	60.25
	Sample 6	20.028	20.012	19.944	7993.562	38.533	4.82	61.02
Series 3	Sample 7	19.929	20.217	19.837	7992.418	39.070	4.89	61.88
	Sample 8	19.834	20.228	19.9	7983.923	39.746	4.98	63.02
	Sample 9	19.954	20.204	20.007	8065.834	39.927	4.95	62.66
SD							3490.93	
Average								61.21

**Table 19:** Relative density of samples after sintering.

		X [mm]	Y [mm]	Z [mm]	V [mm3]	Weight [g]	Density [g/cm^3^]	Relative Density [%]
Series 1	Sample 1	17.222	17.473	16.186	4870.691	37.901	7.78	98.50
	Sample 2	17.217	17.441	16.258	4881.980	38.416	7.87	99.61
	Sample 3	17.35	17.554	16.409	4997.556	39.082	7.82	98.99
Series 2	Sample 4	17.157	17.425	15.914	4757.661	35.560	7.47	94.61
	Sample 5	17.246	17.272	16.046	4779.669	37.186	7.78	98.48
	Sample 6	17.275	17.315	16.26	4863.636	38.207	7.86	99.44
Series 3	Sample 7	17.367	17.577	16.246	4959.250	39.106	7.89	99.81
	Sample 8	17.407	17.627	16.266	4990.949	39.227	7.86	99.49
	Sample 9	17.439	17.697	16.274	5022.449	39.417	7.85	99.34
SD							4436.13	
Average								98.70

**Table 20:** Estimated cost of production.

CapEx	0.05 €
Material	2.20 €
Other	1.95 €
Total cost of 1 part	4.20 €

## Data Availability

The original contributions presented in the study are included in the article, further inquiries can be directed to the corresponding author.
